# Evaluation of preoperative antibiotic prophylaxis in clean-wound general surgery procedures: a propensity score-matched cohort study at a regional hospital

**DOI:** 10.1186/s12893-024-02616-8

**Published:** 2024-10-07

**Authors:** Mai Charernsuk, Suppadech Tunruttanakul, Leenawat Jamjumrat, Borirak Chareonsil

**Affiliations:** Department of Surgery, Sawanpracharak Hospital, 43 Atthakawee Road, Muang, Nakhon Sawan, 60000 Thailand

**Keywords:** Antibiotic prophylaxis, Surgical wound infection, Surgical wound, General surgery

## Abstract

**Background:**

The administration of antibiotic prophylaxis for clean-wound surgeries is controversial among surgeons, despite guidelines suggesting its use. This study aimed to evaluate its effectiveness in preventing surgical site infections (SSIs) in clean-wound surgeries within a regional setting with varied practices regarding prophylaxis.

**Materials and methods:**

This retrospective cohort study included four types of common general surgeries performed from March 2021 to February 2023 at a tertiary regional hospital in Thailand. The surgeries included skin/subcutaneous excision, thyroidectomy, inguinal hernia repair, and breast surgeries, all of which required regional or general anesthesia. Antibiotic prophylaxis was administered at the discretion of the attending surgeons. SSI diagnosis followed standard diagnostic criteria, involving reviewing medical records and the records of the infection control unit. Infection risk factors were examined. The primary outcome comparison used inverse probability treatment weighting of propensity scores, with covariate balance evaluated.

**Results:**

Of the 501 surgeries identified, 84 were excluded, leaving 417 eligible for analysis. Among these patients, 233 received prophylactic antibiotics, for an SSI rate of 1.3%, while 184 did not receive antibiotics, for an SSI rate of 2.2%. A comparative analysis using propensity score weighting revealed no statistically significant difference in the incidence of SSI between the groups (risk ratio [95% confidence interval]: 0.54 (0.11, 2.50), *p* = 0.427).

**Conclusion:**

In this practical setting, with the given study size, antibiotic prophylaxis in common general surgeries involving clean wounds did not significantly prevent SSIs. Routine use recommendations should be re-evaluated.

**Trial registration:**

Not applicable as this study is a retrospective cohort study and not a clinical trial.

## Introduction

Surgical site infections (SSIs) are significant complications of surgery, leading to adverse outcomes such as prolonged hospitalization, the need for additional surgical interventions, and, in severe cases, mortality [1]. The risk of SSIs is influenced by various factors, including the patient’s immune response, the extent of the surgical procedure, and the type of surgical wound [2]. Among these, clean wounds typically pose the lowest risk of infection [3].

Preoperative antibiotic prophylaxis is a crucial strategy for reducing the risk of SSIs [4]. However, its effectiveness in patients undergoing less invasive procedures involving clean wounds is debated among surgeons. While some meta-analyses suggest a reduction in SSI risk with the use of preoperative antibiotics, these studies often include data from clean-contaminated wound procedures [5, 6]. Additionally, the effectiveness of antibiotics may vary depending on the specific body part involved [7]. Consequently, some surgeons have adopted the practice of omitting routine antibiotic prophylaxis for clean wound surgeries [8, 9]. Although many surgeons in our country followed national guidline recommending routine antibiotic prophylaxis in some clean wound surgeries (e.g. non-reconstructive breast cancer procedures, hernia [abdomen/ groin], cancer related head and neck surgeries, and etc.) [10]. A survey conducting in our department reveal difference arroach raging from routinely prophylaxis, selective prophylaxis (having risk factors such as diabetes mellitus or the use of prothesis), to no antibiotics prescribed in every patient, which reflected mentioned before controversy.

Therefore, our study aimed to evaluate the effectiveness of preoperative antibiotic prophylaxis in patients undergoing common, less invasive clean-wound surgeries. This research intends to provide a more comprehensive understanding of the role of antibiotics in preventing SSIs in these specific surgical contexts.

## Materials and methods

The data for this study were collected retrospectively from a cohort of patients over two years, from March 2021 to February 2023, at Sawanpracharak Hospital, a tertiary government hospital in the lower northern area of Thailand. All the data were retrieved from the hospital’s electronic data archiving system. We adhered to the confidentiality principles outlined in the Helsinki Declaration, ensuring that all participants remained anonymous. No identifiable individual data were used in our analysis. The study protocol was approved by the Sawanpracharak Hospital Ethical Committee for Research in Human Subjects (COA.25/2023). The requirement for patient consent was waived due to the retrospective study design and use of deidentified data.

In this study, we included (inclusion criteria) data from four common elective general surgery procedures—skin/subcutaneous excision, thyroid surgery, inguinal hernia surgery (with and without mesh reinforcement), and breast surgery (mastectomy or modified radical mastectomy)—that required regional or general anesthesia. Notably, our surgical department does not perform early implantation in breast surgeries. As a result, all mastectomy or modified radical mastectomy cases in our data were conducted without the use of prostheses. For exclusion criteria, we excluded data from patients under 18 years old, minor procedures performed under local anesthesia, patients undergoing emergency surgeries, those with incomplete data, and cases where preoperative antibiotic prophylaxis was considered inappropriate. Additionally, we excluded cases where antibiotic prophylaxis was provided using oral antibiotics, as this practice, although common, is not recommended in our national guidelines [10].

The primary intervention studied was the administration of prophylactic antibiotics. The decision to administer prophylactic antibiotics in this study was made by the attending surgeons (as mentioned in the survey in the introduction section). Some surgeons in our country omit antibiotic prophylaxis for clean-wound surgeries, despite national guidelines. This practice is not considered malpractice and is openly discussed within the College of Surgeons. The decision to use prophylaxis often depends on surgeon preference or specific patient factors (e.g., diabetes or the use of prostheses), rather than strict adherence to guidelines. We believe that surgeons’ preferences for a particular approach may be influenced by their experience, training institutions, or insights shared with colleagues. Appropriate intravenous antibiotic prophylaxis was according to the guideline, that given withing 60 min before a surgical incision. The types of antibiotics were collected. Notably, our hospital does not practice local administration of antibiotics [4]. All surgeries, whether or not they received antibiotic prophylaxis, adhered to the hospital’s standard sterile protocol, which is routinely followed by scrub nurses. This protocol included measures such as advising patients to shower before surgery, wear clean clothes, undergo hair removal if indicated, use traditional skin preparation techniques (e.g., scrub-and-paint [usually with povidone-iodine solution, but also with chlorhexidine gluconate and alcohol when requested]), and proper hand preparation and gloving by the staff. Reusable surgical drapes were used, and no specialized dressings or impregnated drapes were commonly applied. Additional procedures may be required based on individual patient needs, such as recommending smoking cessation, discontinuing certain medications, and other necessary interventions.

Factors associated with surgical infection were reviewed and collected [2, 11]. These included patient age, sex, body mass index (categorized as ≥ 27.5 kg/m^2^, the Asian obesity cutoff value [12]), presence of diabetes mellitus, active smoking within six months before surgery, American Society of Anesthesiology (ASA) status, surgery duration (in minutes), and estimated blood loss. ASA status and estimated blood loss (assessed using visual estimation methods) were determined by anesthesiologists. Other data collected included the incidences of morbidities and mortalities and follow-up status. Some patients may have undergone more than one eligible surgery within the two-year study period (e.g., initial breast excision followed by breast cancer surgery, ipsilateral thyroid surgery followed by contralateral thyroid surgery, or ipsilateral inguinal hernia surgery followed by contralateral inguinal hernia surgery). Therefore, each surgical occasion rather than each individual patient was treated as a separate unit of analysis.

The main outcome measure was the occurrence of SSIs. The diagnostic criteria for superficial SSIs included the following [13]: 1) purulent drainage from the superficial incision, 2) positive organism growth from aseptically obtained fluid or tissue culture, 3) surgical wound exploration with or without positive culture, or 4) diagnosis of incisional wound infection by the surgeon. For deep SSIs, the diagnostic criteria were as follows [13]: 1) purulent drainage from the deep incision not involving organ/space components, 2) spontaneous or deliberate opening of a deep incision with culture-positive findings and accompanying symptoms (fever, localized pain, or tenderness), 3) identification of abscess or infection in the deep incision during reoperation or through histopathologic or radiologic examination, or 4) diagnosis of deep incisional SSI by a surgeon or attending physician. Additionally, the incidence of SSI was cross-checked with data from the hospital’s infection control unit during the study period. All SSI diagnoses had to be made within 30 days postoperation (or up to one year for patients with mesh implants). The secondary outcome measure included all complications identified within 30 days postoperation (during hospital admission or the follow-up period). Overall complication grading was classified using the Clavien–Dindo classification system [14]. Additionally, whether the complication was surgical or medical was also recorded. SSIs were also included and graded as part of the overall complication assessment.

Patients were followed according to our department’s protocol tailored to specific disease conditions. All four types of surgeries included in the study were followed up at approximately one month postoperation. Patients who underwent inguinal hernia repair underwent additional follow-up at six to twelve months. For thyroid and breast surgeries, follow-up depended on pathological results, with more frequent visits scheduled after the initial one-month appointment.

The study sample size was calculated based on the assumption of reducing the incidence of SSI from approximately 10% (based on both the hospital’s historical average incidence (10%) and the literature (7.4%) [5]) to approximately one-third, or approximately 3%. With a power of 0.8, an alpha error of 0.05, and a one-sided test, the calculated sample size was approximately 360.

The variables are reported as the means ± standard deviations or medians with interquartile ranges for continuous variables and as numbers with proportions for categorical variables. For univariable comparisons, t tests or Mann‒Whitney U tests were used for continuous data, and Fisher’s exact tests were used for categorical data.

### Propensity score matching

To mitigate bias in this observational study, the primary outcome (SSI) was analyzed using propensity scores with inverse probability treatment weighting analysis [15]. The factors likely influencing SSI risk, included in the propensity score calculation, were patient age, sex, body mass index, active smoking status, diabetes mellitus status, ASA III status, type of surgery categorized by mean operative time, estimated blood loss, and use of mesh. Four types of surgeries were planned to be categorized according to their incidence of SSI based on data from this study. Propensity scores were generated using logistic regression.

Inverse-probability treatment weighting was employed to efficiently preserve sample sizes. Patients receiving prophylactic antibiotics were weighted by the inverse of their propensity score, while those not receiving antibiotics were weighted by the inverse of one minus the propensity score. Covariate balance after weighting was assessed using mean standardized differences and Kernel density plots [15, 16].

The estimated treatment effect on the SSI was calculated using binary regression (generalized linear models for the binomial family). A *p* value of < 0.05 was considered to indicate statistical significance. Missing data were managed according to type and proportion of the missing.

Sensitivity analysis was planned to test the impact of varying determinant factors on outcome variability. These sensitivity analyses addressed missing values (analyzed according to the missing data management method), excluded patients who used a prosthesis (mesh), which typically warrants prophylactic antibiotics to clarify the utility of prophylactic antibiotics in general cases, and evaluated SSI outcomes based on each type of surgery. All sensitivity analyses were conducted using the same statistical procedures.

## Results

**Fig. 1:**
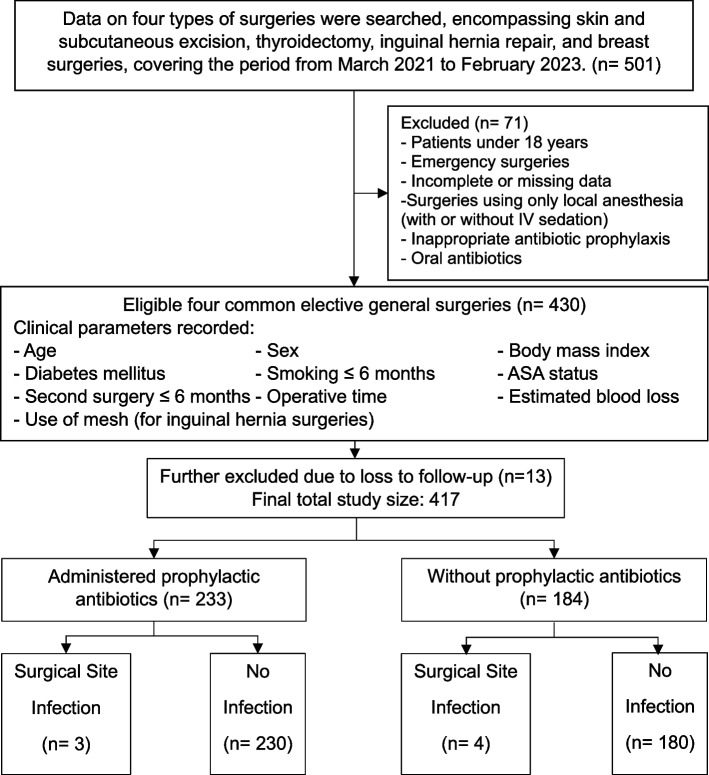
Study participant flow diagram. ASA: american society of anesthesiology

The flow of study participants is depicted in Fig. 1. During the cohort period, data from 501 surgeries were obtained. Of these, 71 surgeries were excluded based on the exclusion criteria, leaving 430 eligible surgeries for analysis. Our data showed a 3% loss to follow-up (13 patients), with no missing determinant data. These incomplete follow-ups were excluded from the analysis (complete case analysis) as they could affect the SSI diagnostic criteria, and a loss of under 5% introduces minimal bias [17]. However, data from one patient was included despite not meeting the follow-up length criteria, as the patient passed away during the surgical admission. A total of 417 patient data were included in the main outcome analysis. Of these, 233 received prophylactic antibiotics (ATB group), for an SSI rate of 1.3% (3 patients). The remaining 184 surgeries did not receive antibiotic prophylaxis (No ATB group), with an SSI rate of 2.2% (4 patients).

Table 1 presents the demographic details of the patients. The patient population was generally older and predominantly female. The use of antibiotic prophylaxis was imbalanced among the types of surgeries, particularly hernia surgeries. This imbalance was largely due to physicians’ preference to administer antibiotics when using mesh; 78.0% (71 out of 91) of inguinal hernia repairs with mesh received antibiotics. In the ATB group, the majority of patients (224, 96.1%) received first-generation cephalosporin (Cefazolin) intravenously. Clindamycin was administered to 6 patients (2.6%) due to suspected penicillin allergies. Third-generation cephalosporin (Ceftriaxone) was given to 3 patients (1.3%).

**Table 1:** Participant characteristics

	Pre-operative antibiotic prophylaxis (*n* = 417)		
Patient characteristic, n (%)	Not Received (*n* = 184)	Received (*n* = 233)	*p*-value
Age (years, Mean ± SD)	55.0 ± 14.4	57.8 ± 15.0	0.058
Male	51 (27.7)	104 (44.6)	0.001
BMI (Mean ± SD)	24.1 ± 4.5	23.8 ± 4.1	0.546
BMI ≥ 27.5 kg/m^2^	35 (19.0)	47 (20.2)	0.805
Active smoking	20 (10.9)	36 (15.5)	0.194
Diabetes Mellitus	32 (17.4)	36 (15.5)	0.596
ASA status III	56 (30.4)	78 (33.5)	0.528
Type of surgery			< 0.001
- Skin/ Subcutaneous	10 (5.4)	27 (11.6)	
- Inguinal hernia^a^	47 (25.5)	101 (43.4)	
Using mesh	20 (10.9)	71 (30.5)	
- Thyroid	52 (28.3)	28 (12.0)	
- Mastectomy or MRM	75 (40.8)	77 (33.1)	
Estimated blood loss (ml, Mean ± SD)	51.9 ± 64.6	47.0 ± 62.4	0.437
Blood Transfusion	2 (1.1)	0 (0.0)	0.194
Operative time (minutes, Mean ± SD)	73.0 ± 37.4	73.0 ± 44.4	0.988
Complications	27 (14.7)	19 (8.2)	0.041
Surgical site infection	4 (2.2)	3 (1.3)	0.704

*ASA* American Society of Anesthesiology, *BMI* Body Mass Index, *MRM* Modified Radical Mastectomy, *SD* Standard Deviation

^a^Using mesh 91 (61.5%): Received antibiotics prophylaxis in 71 cases (78.0%)

**Fig. 2:**
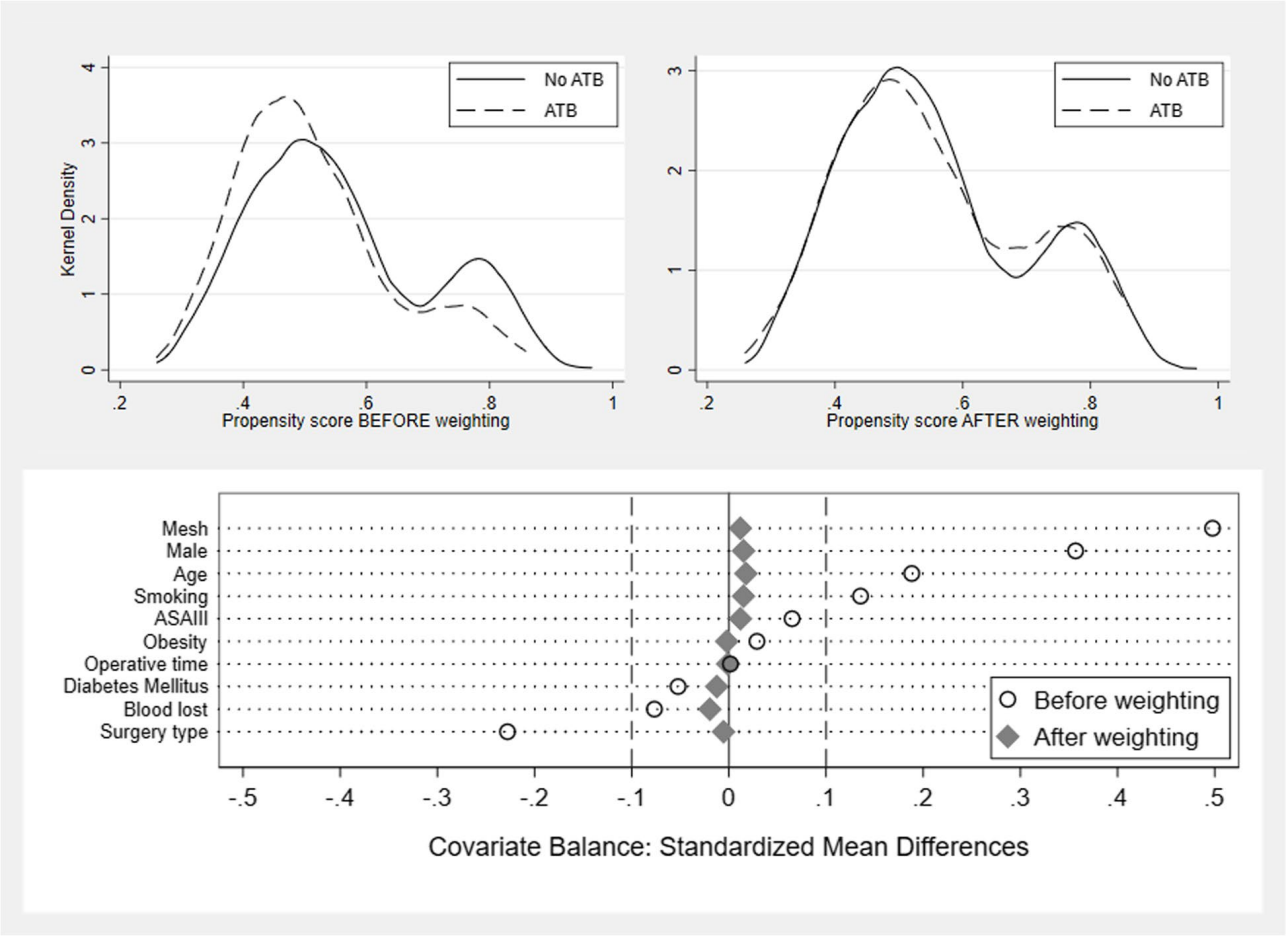
Covariate balance before and after propensity score weighting, shown with kernel density plots (above) and comparison of mean standardized differences (below). Obesity: Body mass index ≥ 27.5 kg/m^2^. ASA: American Society of Anesthesiology; ATB: Antibiotic prophylaxis administered

Propensity scores were generated as described in the methods section, with the four types of surgeries categorized into a ternary predictor based on their incidence of SSI. Skin/subcutaneous excision was assigned code 1 (0% SSI), inguinal hernia and thyroid surgeries were assigned code 2 (0.7% and 1.3% SSI, respectively), and mastectomy or modified radical mastectomy was assigned code 3 (3.3% SSI). After propensity score weighting, the covariate balance was evaluated by comparing the mean standardized differences and Kernel density plots, as shown in Fig. 2. All covariates had an acceptable range of mean standardized differences after weighting (within 10%), compared to some covariates exceeding 10% before weighting. The Kernel density plots for the weighted and unweighted two-treatment groups (ATB group vs. No ATB group) also showed better alignment in the after-weighted plots.

Table 2 presents the main results and SSI incidence by type of surgery. Mastectomy or modified radical mastectomy had the highest SSI rate (3.3%), while no SSIs were observed in skin/subcutaneous excisions. Additionally, the SSI incidence for each surgery type was comparable between the ATB and No ATB groups, with no significant differences observed. When comparing the groups using propensity score weighting, no statistically significant difference in the incidence of SSI or complication rate between the ATB group and the No ATB group. The risk ratio (95% confidence interval [CI]) for SSIs was 0.54 (0.11, 2.50), with a *p* value of 0.427. The risk ratio for complications was 0.62 (0.35, 1.10), with a *p* value of 0.103.

**Table 2:** Incidence of surgical site infections by type of surgery and comparative results of surgical site infection and complication rates between patients receiving and not receiving antibiotic prophylaxis, including sensitivity analyses (total *n* = 417)

**Surgical Site Infection by Type of Surgery and Preoperative Antibiotic Prophylaxis** (*n* = 417)					
	Not Received (*n* = 184)		Received (*n* = 233)		*p*-value*
Type of surgery	SSI	No SSI	SSI	No SSI	
- Skin/ Subcutaneous (*n* = 37), n (%)	0 (0.0)	10 (100.0)	0 (0.0)	27 (100.0)	NA
- Inguinal hernia^a^ (*n* = 148), n (%)	0 (0.0)	47 (100.0)	1 (0.9)	100 (99.1)	1.000
No mesh (*n* = 57), n (%)	0 (0.0)	27 (100.0)	0 (0.0)	30 (100.0)	NA
Using mesh (*n* = 91), n (%)	0 (0.0)	20 (100.0)	1 (1.4)	70 (98.6)	1.000
- Thyroid (*n* = 80), n (%)	1 (1.9)	51 (98.1)	0 (0.0)	28 (100.0)	1.000
- Mastectomy or MRM (*n* = 152), n (%)	3 (4.0)	72 (96.0)	2 (2.6)	75 (97.4)	0.679
**Primary Comparative Outcomes** (*n* = 417)^a^		Risk Ratio (95% Confidence interval)		Standard Error	*p*-value**
- Surgical site infection		0.54 (0.11, 2.50)		0.42	0.427
- Complications		0.62 (0.35, 1.10)		0.18	0.103
**Sensitivity Analyses:** Surgical Site Infection					
- Total cohort (*n* = 430)^b^		0.48 (0.10, 2.26)		0.38	0.353
- Excluding surgeries using mesh (*n* = 326)		0.38 (0.07, 2.11)		0.33	0.268
- Only Mastectomy or MRM (*n* = 152)^c^		0.27 (0.04, 1.90)		0.27	0.187

*NA* Not Applicable due to no events occurring in either group, *MRM* Modified Radical Mastectomy, *SSI *Surgical Site Infection

^*^p-value from Fisher’s exact test; **p-value from binary regression using Inverse-Probability Treatment Weighting of Propensity Scores

^a^The outcomes exclude 13 (3%) patients who did not complete the 30-day follow-up (or one-year follow-up for patients using mesh), violating the surgical site infection diagnosis criteria

^b^Sensitivity analysis includes the 13 (3%) patients who did not complete follow-up

^c^Sensitivity analysis includes only mastectomy or modified radical mastectomy patients (excluding other types of surgery), notably the effect size of other surgery types cannot be calculated due to no events occurring in one or both groups

In the No ATB group, 27 (14.7%) complications occurred, graded as follows: 1, Grade V (death) due to disease pathology (anaplastic thyroid carcinoma leading to airway obstruction after thyroid surgery); 2 Grade IV; 4 Grade III; 7 Grade II; and 13 Grade I. In the ATB group, there were 19 (8.2%) complications, graded as follows: 5 Grade III; 4 Grade II; and 10 Grade I, with no mortality. Most complications were unrelated to surgical site infections and were primarily related to bleeding complications. Seroma was the most common Grade I complication (78.3%). Medical complications occurred in two patients (4.3%): one with gouty arthritis and another who survived post-operative multiorgan failure.

Table 2 also presents the sensitivity analysis results. The first analysis addressed the 13 patients who did not complete follow-up (missing certified SSI outcomes). It is possible that these patients did not return due to the absence of problems or infections after surgery. Therefore, incomplete follow-up outcomes were handled by re-intoducing to the data and analysis (*n* = 430). The results were similar to those of the main analysis (complete case analysis [*n* = 417]), showing no difference in SSI rates between the ATB and No ATB groups. The second sensitivity analysis involved removing data on surgeries that used mesh. The SSI rates remained comparable between the two groups. Lastly, when including only mastectomy or modified radical mastectomy cases (*n* = 152), the SSI risk ratio remained similar between the two groups. The effect size for other surgery types cannot be calculated due to no events occurring in one or both groups.

## Discussion

Our results revealed a low incidence of SSIs in common general surgeries involving clean wounds, at 1.7% (7 cases). Even though our hospital is located in a tropical area where the likelihood of infection is relatively high [18], the incidence of SSIs in clean wounds remained low. This incidence also reflects the practical setting, as our hospital is a regional hospital. The low incidence observed aligns with other reported SSI incidences in clean wounds, which are approximately 3% [19, 20].

Given such a low incidence of SSI, the benefit of applying antibiotic prophylaxis might be minimal. This was confirmed by our comparative results, which showed no significant difference in the incidence of SSI between patients receiving and not receiving antibiotic prophylaxis (risk ratio [95% CI]: 0.54 (0.11, 2.50), *p* value 0.427).

Guidelines, including those from Thailand, recommend routine antibiotic prophylaxis for clean-wound surgeries [10, 21–23]. However, in practice, some surgeons disregard these recommendations and either omit antibiotic prophylaxis entirely for clean-wound surgeries [9] or selectively administer it only to higher-risk patients (e.g., those with certain risk factors or those using prostheses). Our study clearly illustrates these varied approaches, reflecting the diverse practices of surgeons at our institute.

This discrepancy in routine antibiotic prophylaxis for clean-wound surgeries is further highlighted by contrasting evidence. Despite published meta-analyses (the highest level of evidence) supporting its use [5, 6], there are also observational studies (reflecting real-world settings) that report contrary findings [8, 9, 24]. From our study’s outcome perspective—showing a low SSI rate and no significant difference in SSI rates between groups—routine antibiotic prophylaxis may not be justified.

The selective administration of prophylaxis for at-risk patients might be more reasonable, although this is beyond the scope of our study. Additionally, other prophylactic interventions, such as surgical team hand preparation, the use of powder-free gloves, and proper patient skin preparation, might offer more benefits [21, 23]. Avoiding unnecessary antibiotic use can reduce drug side effects, resistance, and allergies and can also be more cost-effective [25, 26]. One large retrospective cohort study also detected an association between the use of preoperative antibacterial prophylaxis in surgeries such as abdominal hysterectomy, hip arthroplasty, craniotomy, and colon, cardiac, or vascular surgery, and *Clostridium difficile* infection, a significant healthcare-associated infectious complication [27].

The strengths of our study lie in its reflection of real-world practices [28], where various surgeons managed their common clean-wound surgeries. These approaches range from strictly following national guidelines and routinely administering prophylactic antibiotics to selectively applying them or completely disregarding their use.

However, there were some limitations to our study. First, our sample size was based on an estimated 10% infection rate, but the actual infection rate in our study was only 1.6%. This overestimated SSI rate was partly based on a combination of SSI rates from both clean and clean-contaminated surgeries reported in the literature (7.4%) [5], as well as our hospital’s historical data (around 10%). The latter was somewhat unreliable due to the lack of a standardized data collection protocol. Additionally, the ATB group showed a trend toward a lower incidence of SSI (Risk ratio [95% CI]: 0.54 [0.11, 2.50]). With a larger sample size, antibiotic prophylaxis may significantly reduce the risk of SSI. Despite this, we collected data from common general surgeries over two years. We believe that if the prophylaxis effect was not noticeable within this period, its effectiveness is likely too low to warrant routine use. Moreover, comparing very low infection rates would necessitate a very large sample size. For example, reducing the SSI rate from 1.6% (as observed in our study) to 0.5% (a reduction to one-third) would require approximately 2,694 participants. Conducting such a large-scale trial, which would provide higher-level evidence, may be nearly impossible due to significant logistical and ethical challenges. The second limitation pertains to our study design. Retrospective data collection has inherent limitations regarding the completeness and reliability, including inconsistent inter-rater reliability due to subjective recording by healthcare providers, particularly concerning the outcome [28, 29]. Additionally, unmeasured confounders present a significant limitation in this observational study compared to a clinical trial (with randomization), particularly with respect to confounding by indication—where healthcare providers tend to assign treatment (in this case, prophylactic antibiotics) to specific patients [30]. Mild SSIs in healthy individuals might also resolve on their own [26]. However, since our patients were from a regional area, they typically returned to the hospital when adverse outcomes occurred, lending credibility to our study outcomes. The possibility of self-resolved SSIs also suggests that prophylactic antibiotics may not always be necessary. Furthermore, although the study design significantly affected the reliability of our findings, conducting a large clinical trial in this area, as mentioned before, would be impractical and, in some cases, unethical. Third, one of our SSI diagnosis criteria relied on the decision of a surgeon or attending physician, which can introduce subjectivity. The Centers for Disease Control and Prevention (CDC) guidelines suggest that SSI diagnosis should be supported by microbiological evidence or imaging whenever possible [22]. Alternatively, using more objective diagnostic criteria, such as the ASEPSIS score [31], could improve the clinical relevance and objectivity of the diagnosis. Lastly, because our observed SSI rate was exceptionally low, our results may not be generalizable to populations with higher SSI rates due to possible differences in risk factor profiles (e.g., higher rates of active smoking or obesity). The SSI rate at each center should be evaluated before these results are applied.

## Conclusions

In this practical setting, with this study size, antibiotic prophylaxis in common clean-wound general surgeries did not demonstrate a significant improvement in preventing SSI compared to not applying prophylaxis, particularly in institutes with low SSI rates. The routine use of antibiotic prophylaxis should be re-evaluated, considering its limited effectiveness in such settings.

## Data Availability

No datasets were generated or analysed during the current study.

## List of abbreviations

ASA: American society of anesthesiology
SSI: Surgical site infection
CI: Confidence interval
